# Supplementary material for: Antiplatelet and Antithrombotic Effects of Isaridin E Isolated from the Marine-Derived Fungus via Downregulating the PI3K/Akt Signaling Pathway
Source: Mar Drugs. 2021 Dec 24;20(1):23. doi: 10.3390/md20010023 (PMC8780978; doi:10.3390/md20010023)
Supplement: Supplementary file 1 [file marinedrugs-20-00023-s001.zip › marinedrugs-1494981-supplementary.pdf]

Supporting Information

Antiplatelet and antithrombotic effects of isaridin E isolated from the marine-derived fungus via down-regulating PI3K/Akt signaling pathway

Ni Pan[1,2], Zi-Cheng Li[1], Zhi-Hong Li[1], Sen-Hua Chen[3], Ming-Hua Jiang[3], Han-Yan Yang[1], Yao-Sheng Liu[1], Rui Hu[1], Yu-Wei Zeng[1], Le-Hui Dai[4], Lan Liu[3,*], and Guan-lei Wang[1,*]

[1] Department of Pharmacology, Zhongshan School of Medicine, Sun Yat-sen University, Guangzhou, 510080, China.
[2] Institute of Pediatrics, Guangzhou Women and Children's Medical Centre, Guangzhou Medical University, Guangzhou, 510080, China.
[3] School of Marine Sciences, Sun Yat-sen University, Guangzhou 510006, China.
[4] Department of Basic Medical Sciences, Zhongshan School of Medicine, Sun Yat-sen University, Guangzhou, 510080, China.
[*] Correspondence: wangglei@mail.sysu.edu.cn (W.G.L); cesllan@mail.sysu.edu.cn (L.L). Tel. : +86-20-87330300.

Contents

Figure S1. The HR-ESIMS spectrum of compound 1. 2

Figure S2. The ^{1}H NMR (400MHz) spectrum of compound 1 in $CDCl_3$ 2

Figure S3. The ^{13}C NMR (100MHz) spectrum of compound 1 in $CDCl_3$. 3

Table S1. Effects of isaridin E on the hematologic parameters. 3

Table S2. Effects of isaridin E on the coagulation parameters. 4

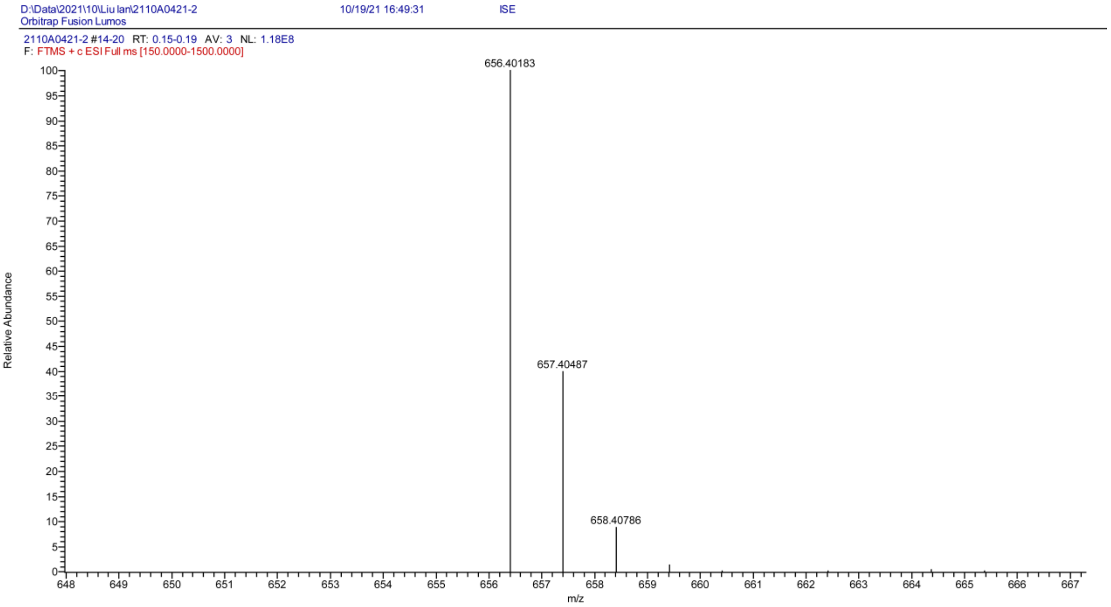

SPECTRUM - simulation m/z	Theo. Mass	Delta (ppm)	RDB equiv.	Composition
656.40183	656.40178	0.08	11.5	$C_{35}H_{54}O_7N_5$

Figure S1. The HR-ESIMS spectrum of compound 1 (isaridin E).

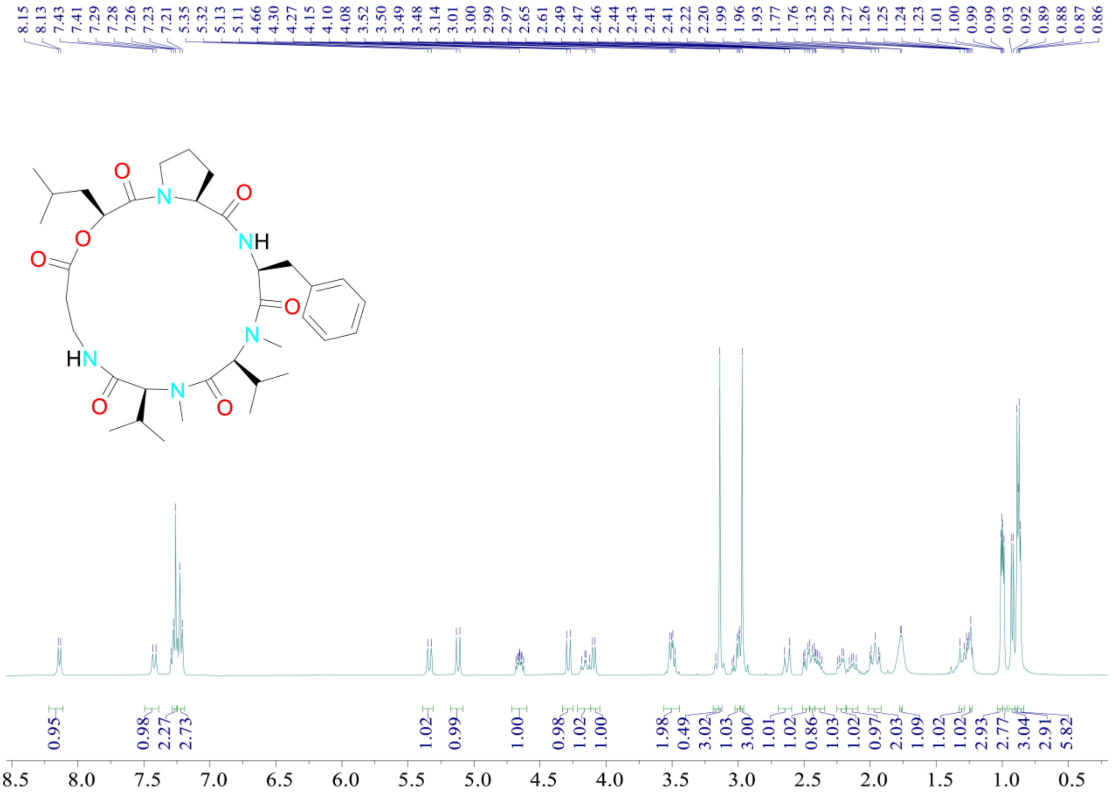

Figure S2. The ^1H NMR (400MHz) spectrum of compound 1 (isaridin E) in $CDCl_3$.

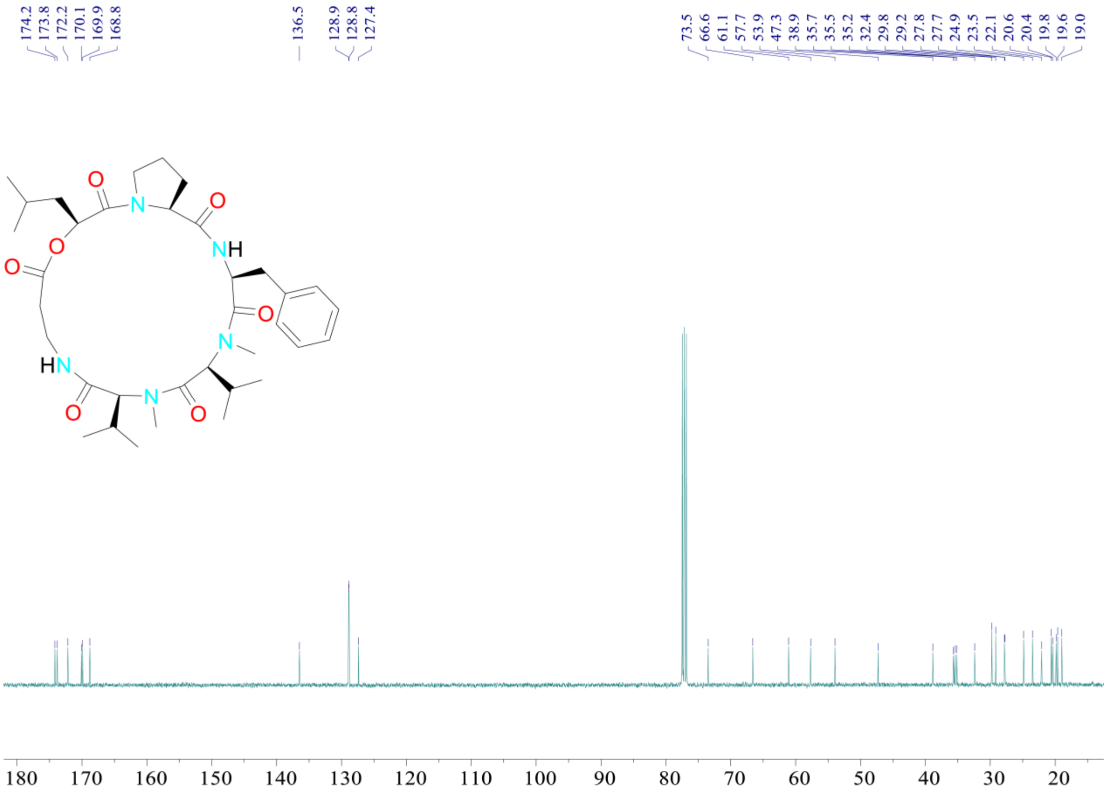

Figure S3. The ^{13}C NMR (100MHz) spectrum of compound 1 (isaridin E) in $CDCl_3$.

Table S1. Effects of isaridin E on the hematologic parameters.

	NS	Vehicle	Isaridin E	*P*[1] value	*P*[2] value
PLT (10^9/L)	1303 ± 114	1267 ± 77	1233 ± 65	0.8010	0.6100
MPV (fL)	5.1 ± 0.3	4.9 ± 0.3	5.3 ± 0.3	0.6763	0.7896
WBC (10^9/L)	3.3 ± 0.6	2.8 ± 0.4	3.3 ± 0.5	0.4372	0.9799
LY % (%)	84.2 ± 2.2	82.3 ± 3.0	83.1 ± 2.5	0.6325	0.7407
MO % (%)	2.9 ± 0.7	3.4 ± 0.7	2.7 ± 0.6	0.6400	0.8544
NE % (%)	13.1 ± 2.1	14.2 ± 2.0	14.8 ± 2.2	0.7200	0.5984
RBC (10^12/L)	8.0 ± 0.4	7.6 ± 0.6	7.8 ± 0.3	0.5521	0.6919
HCT (%)	39.2 ± 0.8	38.3 ± 1.2	39.5 ± 1.4	0.5518	0.8662
MCV (fL)	51.4 ± 0.7	52.1 ± 0.6	51.1 ± 0.8	0.4805	0.7655
MCH (pg)	17.1 ± 0.5	17.5 ± 0.6	17.3 ± 0.4	0.6161	0.7695
MCHC (g/L)	328 ± 8	325.8 ± 5	330 ± 8	0.8287	0.8616
RDW (%)	15.6 ± 0.4	15.9 ± 0.5	15.2 ± 0.5	0.6035	0.5828

Note: Mice were given a single dose of normal saline (NS), vehicle solution (Vehicle) or isaridin E (100 mg/kg) respectively by gavage for 3 h, and then the hematologic parameters were measured. *P*[1], NS *vs* Vehicle; *P*[2], NS *vs* isaridin E, n=5 mice for each group. PLT, platelet count; MPV, mean platelet volume; WBC, white blood cell; LY %, lymphocyte percentage; MO %, monocyte percentage; NE %, neutrophil percentage; RBC, red blood cell; HCT, hematocrit; MCV, mean red blood cell volume; MCH, mean red blood cell hemoglobin; MCHC, mean red blood cell hemoglobin concentration; RDW, red cell distribution width.

Table S2. Effects of isaridin E on the coagulation parameters.

	ATPP (s)	PT (s)	TT (s)	FIB (g/L)
NS	30.5 ± 1.5	12.5 ± 0.4	14.6 ± 0.5	3.2 ± 0.1
Vehicle	31.3 ± 1.1	11.8 ± 0.3	15.0 ± 0.6	3.3 ± 0.2
Isaridin E	32.4 ± 1.0	12.1 ± 0.4	14.0 ± 0.8	3.1 ± 0.1
P^1 value	0.6496	0.2415	0.5909	0.7135
P^2 value	0.2924	0.5349	0.5425	0.4417

Note: 3 h after mice were administrated with a single dose of saline (NS), vehicle solution (Vehicle) or isaridin E (100 mg/kg) by oral gavage, coagulation parameters were analyzed. P^1, NS *vs* Vehicle; P^2, NS *vs* isaridin E, n = 5 mice/group. APTT, activated partial thromboplastin time; TT, thrombin time; FIB, fibrinogen; PT, prothrombin time.
